# Infectious serology patterns in clinically diagnosed erythema multiforme

**DOI:** 10.1016/j.jdin.2026.05.003

**Published:** 2026-05-12

**Authors:** Thi Huyen Tran, Van Dan Bui, Anh Duy Dinh

**Affiliations:** a Department of Allergy and Clinical Immunology, Hanoi Medical University, Hanoi, Vietnam; b Faculty of Medicine, Hanoi Medical University, Hanoi, Vietnam

**Keywords:** erythema multiforme, herpes simplex virus, *Mycoplasma pneumoniae*, serology

*To the Editor:* Erythema multiforme (EM) is a mucocutaneous disease commonly associated with infections, particularly herpes simplex virus (HSV), *M pneumoniae* (MP), Epstein–Barr virus (EBV), and cytomegalovirus (CMV).^1^ Previous studies describing the epidemiologic characteristics of EM patients have often been limited to retrospective designs, which may lead to underestimation of the underlying etiologic agents. Here, we present the results of a cross-sectional study in EM patients who underwent IgM and IgG serologic testing for 4 pathogens: HSV, MP, EBV viral-capsid antigen (EBV VCA), and CMV, at the National Hospital of Dermatology and Venereology in Vietnam.

Since histologic confirmation was not routinely performed, the diagnosis was based on clinical assessment. A total of 54 patients clinically diagnosed with EM at our hospital between 2018 and 2024 were included in the study. After the clinical diagnosis had been established, all patients underwent serologic testing for 4 etiologic agents. Among them, 5 (9.2%) were positive for HSV IgM, 8 (14.8%) for MP IgM, and 4 (7.4%) for both HSV and MP IgM. Two patients (3.7%) had equivocal EBV VCA IgM results. One patient each showed positivity for EBV VCA IgM alone, HSV combined with equivocal EBV VCA IgM, HSV + MP + EBV VCA IgM, and HSV + MP + CMV IgM. Thirty-one patients (57.3%) were IgM negative for all 4 pathogens, among whom 2 reported a recent history of herpes infection.

Clinical characteristics are presented in Table I. Most patients in this study experienced their first episode of EM, and a prior history of herpes infection was relatively uncommon. Patients with positive IgM for at least 1 pathogen were younger, more frequently reported prodromal symptoms, and had a lower prevalence of HSV IgG positivity compared with the remaining patients. Clinical characteristics among groups with HSV IgM, MP IgM, and combined HSV + MP IgM positivity is shown in Table II.

**Table I:** Comparison of clinical characteristics among erythema multiforme groups

Characteristics	IgM positive – EM∗ (*n* = 23)	IgM negative - EM (*n* = 31)	*P*-value
Age, mean (SD)	21.3 (14.0)	40.7 (17.8)	<.001
Gender, *n* (%)			
Male	9 (39.1)	14 (45.2)	.658
Female	14 (60.9)	17 (54.8)	
Distribution of skin lesions, *n* (%)			
Acral	5 (21.7)	1 (3.2)	.073
Disseminated	18 (78.3)	30 (96.8)	
Mucosal involvement, *n* (%)	7 (30.4)	6 (19.4)	.346
Mouth	5 (21.7)	4 (12.9)	.472
Ocular	2 (8.7)	0 (0.0)	.177
Genital	3 (13.0)	2 (6.5)	.640
Prodromal symptoms, *n* (%)	11 (47.8)	6 (19.4)	.026
Cough	8 (34.8)	6 (19.4)	.201
Fever	9 (39.1)	2 (6.5)	.005
Recent herpes infection, *n* (%)	0 (0)	2 (6)	.502
EM antecedents, *n* (%)	0 (0)	1 (3)	1.000
IgG positive, *n* (%)			
HSV	16 (69.6)	31 (100.0)	.001
MP	4 (17.4)	7 (22.6)	.741
EBV VCA	22 (95.7)	31 (100.0)	.426
CMV	23 (100.0)	31 (100.0)	
Time from the onset to hospital admission, median (IQR)	5.00 (4.00-7.00)	5.00 (4.00-10.00)	.839
Leukocytosis at admission, *n* (%)	10 (43.5)	12 (38.7)	.724

*CMV*, Cytomegalovirus; *EBV VCA*, Epstein–Barr virus viral-capsid antigen; *EM*, erythema multiforme; *HSV*, herpes simplex virus.

∗ Patients positive for at least 1 pathogen.

**Table II:** Clinical characteristics among IgM positive - erythema multiforme groups

Characteristics	HSV (*n* = 6)	MP (*n* = 8)	HSV + MP (*n* = 6)
Age, mean (SD)	23.0 (10.7)	23.9 (14.0)	14.2 (13.6)
Gender, *n* (%)			
Male	2 (33.3)	3 (37.5)	2 (33.3)
Female	4 (66.7)	5 (62.5)	4 (66.7)
Distribution of skin lesions, *n* (%)			
Acral	1 (16.7)	0 (0.0)	2 (33.3)
Disseminated	5 (83.3)	8 (100)	4 (66.7)
Prodromal symptoms, *n* (%)	2 (33.3)	2 (25.0)	6 (100.0)
Cough	1 (16.7)	2 (25.0)	4 (66.7)
Fever	1 (16.7)	2 (25.0)	5 (83.3)
Mucosal involvement, *n* (%)	1 (16.7)	1 (12.5)	3 (50.0)
Mouth	1 (16.7)	0 (0.0)	2 (33.3)
Ocular	0 (0.0)	1 (12.5)	1 (16.7)
Genital	1 (16.7)	1 (12.5)	0 (0.0)
IgG positive, *n* (%)			
HSV	6 (100.0)	6 (75.0)	2 (33.3)
MP	1 (16.7)	2 (25.0)	1 (16.7)

The accuracy of serology testing should be considered in EM patients. For HSV IgM, serologic testing does not provide information on the HSV type, and the delayed IgM seroconversion during primary infection as well as the limited accuracy during recurrent episodes may affect the results of this test.^2^^,^^3^ In our study, we observed that a positive HSV IgG result may appear to have a stronger influence on HSV IgM results than the interval between symptom onset and serologic testing, as this interval did not differ significantly between the comparison groups. Additionally, MP IgM levels may remain elevated for up to 6 months after the acute episode and therefore may not accurately indicate the causative role of MP in EM.^4^ Multiple IgM seropositivity may indeed occur in patients with EM, which may be partly attributable to MP co-infection with other microbial pathogens.^5^

This study has several limitations, including the lack of HSV PCR, MP respiratory PCR, and confirmatory EBV/CMV testing; limited clinical syndrome characterization; no routine biopsy confirmation; and no recurrence follow-up. MP respiratory PCR results should also be interpreted with caution, given the high prevalence of asymptomatic carriage.^4^

Overall, a considerable proportion of multiple IgM seropositivity was observed among patients with EM. The use of serologic tests to identify the etiologic agent should take into consideration factors related to their accuracy.

### Declaration of generative AI and AI-assisted technologies in the writing process

Artificial intelligence was used for manuscript translation under human supervision.

## Conflicts of interest

None disclosed.
